# Obesity and Dyslipidemia in Chinese Adults: A Cross-Sectional Study in Shanghai, China

**DOI:** 10.3390/nu14112321

**Published:** 2022-05-31

**Authors:** Junjie Zhu, Yue Zhang, Yiling Wu, Yu Xiang, Xin Tong, Yuting Yu, Yun Qiu, Shuheng Cui, Qi Zhao, Na Wang, Yonggen Jiang, Genming Zhao

**Affiliations:** 1 Key Laboratory of Public Health Safety of Ministry of Education, Department of Epidemiology, School of Public Health, Fudan University, Shanghai 200032, China; zhujunjie233@163.com (J.Z.); xiangyuhk2016@163.com (Y.X.); danmoweiliangtx@gmail.com (X.T.); 17211020011@fudan.edu.cn (Y.Y.); qiuyun2018@fudan.edu.cn (Y.Q.); cuishuheng1995@outlook.com (S.C.); zhaoqi@shmu.edu.cn (Q.Z.); na.wang@fudan.edu.cn (N.W.); 2 Department of Epidemiology and Health Statistics, School of Public Health, Dali University, Dali 671000, China; 3 Department of Epidemiology, School of Public Health, Shanxi Medical University, Taiyuan 030001, China; 17111020009@fudan.edu.cn; 4 Songjiang District Center for Disease Prevention and Control, Shanghai 201600, China; aries2119@163.com; 5 Shanghai Institute of Infectious Disease and Biosecurity, School of Life Science, Fudan University, Shanghai 200032, China

**Keywords:** dyslipidemia, body measurements, general obesity, central obesity, Chinese adults

## Abstract

This study examined the association of obesity and dyslipidemia according to body measurements among Chinese adults in Shanghai, a place in the process of rapid urbanization. Using the baseline data of the Shanghai Suburban Adult Cohort and Biobank study (SSACB), the subjects completed questionnaires and physical examinations, and fasting blood was collected for biochemical assays. We estimated the odds ratios (OR) and 95% confidence interval (CI) by multivariable logistic regression. The prevalence was 12.9% and 28.8% in both general and central obesity, respectively. Compared with the non-obese, the general or central obesity participants had a higher level of TC, TG, LDL-C and lower level of HDL-C. The OR (95%CI) for dyslipidemia was 1.79 (1.69–1.91) and 1.91 (1.83–2.00) in general or central obesity, respectively. Positive associations were also observed between obesity and high TC, high LDL-C, low HDL-C and high TG, with the adjusted OR ranging from 1.11 to 2.00. Significant modifying effect of gender, age, hypertension, and diabetes were found in the association of obesity and different forms of dyslipidemia. The findings of our study indicated that participants with obesity, including general or central obesity, have a higher prevalence of dyslipidemia and gender, age, hypertension, and diabetes might be potential modifiers of the association. More effective attention and interventions should be directed to managing body weight to reduce the prevalence of dyslipidemia.

## 1. Introduction

Dyslipidemia is a common metabolism abnormality involving plasma lipids and lipoproteins, categorized by elevated levels of total cholesterol (TC), triglycerides (TG) and low-density lipoprotein cholesterol (LDL-C), and/or decreased levels of high-density lipoprotein cholesterol (HDL-C). It is a dominant cause of morbidity, mortality, and one of the primary independent modifiable factors for cardiovascular diseases [1,2,3], diabetes [4] and stroke [5] in most countries. With rapid socioeconomic growth, improved standard of living and changes in lifestyles, dyslipidemia has been estimated to be about to rise dramatically worldwide in absolute terms [6,7]. In China, dyslipidemia has attracted much attention in recent years, and is inadequately treated and uncontrolled [8].

Globally, the number of obese people has raised seriously, and obesity has turned into one of the most vital public health threats in the last decades. The data in prevalent overweight/obesity show a rise from 13.4% to 26.4% and a rise from 18.6% to 37.4% in prevalent abdominal obesity in Chinese adults [9]. Several studies have examined the prevalence and risk factors of dyslipidemia in China [10,11,12]. These studies have showed that general obesity significantly influences lipid level; meanwhile, the association of obesity with lipid abnormalities depends not only on general obesity, but on central obesity [13]. Considering the increasing prevalence of dyslipidemia and its health burden, there should be a greater focus on the association of obesity with dyslipidemia. However, these studies were conducted in either urban or rural China. The Songjiang and Jiading District of Shanghai is a suburban area with rapid urbanization, and has experienced huge economic development. There has been a significant change in lifestyles in this region, such as westernized diets, sedentary work, and decreased physical activity, all of which are recognized as main risk factors for dyslipidemia [14,15,16]. Furthermore, little research has explored the modification of gender, age, hypertension and diabetes on the association of obesity with different types of dyslipidemia. This study aims to assess the association of obesity with dyslipidemia according to anthropometric indices, and to analyze the potential effect modification on these associations in a Shanghai natural cohort who live in a rapidly urbanizing suburban area.

## 2. Materials and Methods

### 2.1. Study Design and Population

This study was based on the Shanghai Suburban Adult Cohort and Biobank study (SSACB), which is an ongoing large-scale natural cohort study to identify risk factors for chronic noncommunicable diseases in Chinese adults. The SSACB has been described in great details previously [17]. Briefly, recruitment was conducted through multistage cluster sampling. Seven study sites were selected according to their economic status and population, including four communities from Songjiang (Xinqiao, Sheshan, Maogang, and Zhongshan) and three communities from Jiading (Huangdu. Anting, and Huating). From each community, one third of the villages or committees were randomly selected. According to participant willingness, all residents had lived in Shanghai for at least five years; those aged 20 to 74 years were included, while those with disabilities, terminal illnesses, perceptual impairments, or pregnant or lactating women were excluded. During the period April 2016 to October 2017, 44,887 participants were recruited and interviewed. Furthermore, participants who had malignant neoplasms, liver cirrhosis and thyroid diseases (*n* = 3106), extreme values of body mass index (BMI) or waist circumference (WC) (*n* = 490), unreasonable values for energy intake (*n* = 721) or physical activity (*n* = 164) were excluded. Finally, 40,406 subjects were involved in this analysis. 

### 2.2. Physical Examination and Biochemical Assays

Anthropometric measurements (height, weight, WC) were taken two times by licensed physicians in the communities. Blood pressure was measured two times at five minute intervals using a digital sphygmomanometer, calculated by an average of the two measurements. Serum samples (2 mL) were collected into serum separation tubes, on an empty stomach and in the morning. After collection of the serum fraction, it was stored at −80 °C for no longer than 6 h in a freezer and then transported to the DiAn medical laboratory. Assays of serum TC, LDL-C, HDL-C, and TG were performed using enzyme colorimetry. Glycated hemoglobin (HbA1c) was ascertained using high performance liquid chromatography. Fasting plasma glucose (FPG) was determined using the glycokinase method. 

### 2.3. Diagnostic Criteria

BMI was calculated as weight divided by standing height squared (kg/m^2^), and more than 28 kg/m^2^ was described as general obesity; central obesity is described as having a WC of equal or greater than 90 cm in males and equal or greater than 85 cm in females [18]. We excluded participants with a BMI less than 15 or more than 40 kg/m^2^ or WC less than 50 or more than 150 cm from analysis [19]. The diagnostic criteria for dyslipidemia were: TC ≥ 6.22 mmol/L, or LDL-C ≥ 4.14 mmol/L, or HDL-C < 1.04 mmol/L, or TG ≥ 2.26 mmol/L, or a self-reported history of hyperlipidemia [20]. Hypertension was defined as a self-reported history of hypertension, or a documented history of hypertension in the medical record, or having a resting systolic blood pressure (SBP) ≥ 140 mmHg and/or a diastolic blood pressure (DBP) ≥ 90 mmHg [21]. Diabetes was defined by current ADA criteria: fasting plasma glucose (FPG) ≥ 7.0 mmol/L, or glycosylated hemoglobin (HbA1c) concentration ≥ 6.5%, or a self-reported history of diabetes, or a documented history in the medical record [22].

### 2.4. Assessment of Covariates

Structured questionnaires were used to collect the following variables: age, gender, marital status, education level, alcohol consumption, smoking, physical activity, and China Healthy Diet Index (CHDI). The subjects were separated into two categories: <60 and ≥60 years old. Marital status was recorded in two categories: married, or other (unmarried, divorced, widowed, or separated). Education level was recorded as three categories by years of schooling: ≤6 years, 7–12 years, and ≥12 years. Both smoking and alcohol drinking were separated into two categories: never or ever. Physical activity was expressed as the metabolic equivalent of task (MET)-hours/week and durations over 16 h/day were considered implausible [23]. Overall diet quality was assessed by using the CHDI established by the Chinese Center for Diseases Prevention and Control, which has been described previously in detail [24].

### 2.5. Statistical Analysis

Baseline characteristics of all participants were compared according to whether or not they were generally obese or centrally obese. Variables with continuous measurements were presented as mean ± standard deviation (SD) or median and interquartile range (IQR), and categorical variables as frequency (*n*) and proportion (%). The Kolmogorov-Smirnov test was used to determine if the data were normally distributed. Student’s t test or Mann-Whitney U test were conducted to compare the differences of continuous data, and χ^2^ tests for categorical data. The odds ratio (OR) and 95% confidence intervals (CI) for obesity with different types of dyslipidemia were assessed by using multivariate Logistic regression models. A variety of variables were adjusted, including gender, age, marital status, education level, physical activity, alcohol consumption, smoking, diabetes, hypertension, and CHDI. We tested the potential effect modification by adding multiplicative interaction terms in the multivariable logistic regression models and the interaction terms with *p* < 0.05 were considered statistically significant. Stratified analyses were conducted according to age (<60 and ≥60 years), gender, hypertension (yes, no), and diabetes (yes, no), which were potential effect modifiers for the associations. All data analyses were carried out using SAS version 9.4 (Institute Inc., Cary, NC, USA). All *p*-values were 2-tailed, and an alpha-level of 0.05 was considered statistically significant.

## 3. Results

### 3.1. Baseline Characteristics

A total of 40,406 participants included 16,793 males (41.6%) and 23,613 females (58.4%) in our study. The average age was 56 ± 11 years-old, which was higher for general or central obesity subjects than for non-obesity (all *p* < 0.001). The basic characteristics of participants according to general and central obesity are demonstrated in Table 1. The prevalence of general obesity and central obesity were 12.9% and 28.8%, respectively. According to BMI categories, the prevalent obesity in males was higher compared with that in females (13.8% vs. 12.2%), while 29.8% of males and 28.1% of females had central obesity. Those who were exposed to a relatively low level of education were inclined to suffer a higher prevalence of obesity. Participants with obesity had significantly higher prevalence of diabetes and hypertension, compared with the participants without obesity. The mean values of TC, TG, LDL-C, and FPG increased significantly, and the HDL-C level decreased (*p* < 0.001) in both general obesity and central obesity group.

### 3.2. Prevalence of Different Forms of Dyslipidemia

**Figure 1:**
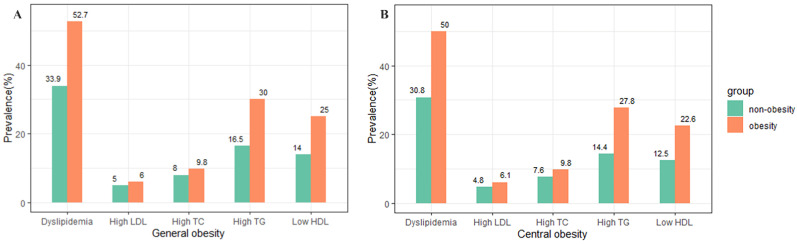
The prevalence of different forms of dyslipidemia categorized by general obesity (**A**) and central obesity (**B**) for all subjects.

The prevalence of different forms of dyslipidemia is shown by BMI and WC categories for all subjects in Figure 1. Compared with non-general obesity, the participants with general obesity had a significantly higher prevalence of different types of dyslipidemia (all *p* < 0.001). The prevalence of high TC, high TG, high LDL-C, low HDL-C, and dyslipidemia were 9.8%, 30.0%, 6.0%, 25.0%, and 52.7% among the participants with general obesity, respectively. The participants with central obesity had a similar higher prevalence of different types of dyslipidemia than those without central obesity (all *p* < 0.001).

### 3.3. Association of Obesity with Different Forms of Dyslipidemia

**Figure 2:**
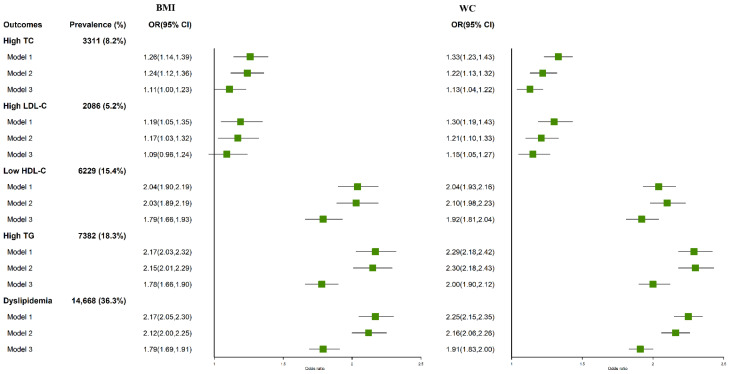
Odds ratios (OR) and 95% confidence intervals (CI) for BMI and WC categories with dyslipidemia. Model 1: unadjusted; model 2: adjusted for gender and age; model 3: additionally adjusted for education level, marriage, physical activity, smoking, alcohol drinking, CHDI, diabetes, and hypertension.

Figure 2 shows the associations of general and central obesity with different types of dyslipidemia. Subjects with obesity either by BMI (OR = 1.79, 95% CI: 1.69–1.91) or by WC (OR = 1.91, 95% CI: 1.83–2.00) had higher risk of dyslipidemia than those without obesity in the multivariable adjusted models. According to BMI categories, general obesity was associated with a 11%, 9%, 78%, and 79% increased risk of high TG, high TC, high LDL-C, and low HDL-C, respectively. According to WC categories, the adjusted OR for high TG, high TC, high LDL-C, and low HDL-C were 2.00 (95% CI: 1.90, 2.12), 1.13 (95% CI: 1.04, 1.22), 1.15 (95% CI: 1.05, 1.27), and 1.92 (95% CI: 1.81, 2.04), respectively, while the OR with high TG for central obesity was greatest.

### 3.4. Stratified Analysis

Stratification by age, gender, hypertension and diabetes suggested that these factors might be potential modifiers on the association between obesity and different types of dyslipidemia (*p* for interaction < 0.05), with few exceptions (Table 2 and Table 3). The associations between general or central obesity and different types of dyslipidemia were statistically significant among the individuals younger than 60 years old, with the OR ranging from 1.23 to 2.13. Compared with 60 years or older, general or central obesity among individuals younger than 60 years old may be associated with a greater risk of different types of dyslipidemia. Males, either with general obesity or central obesity, had significantly increased risk of high TG, TC, LDL-C, low HDL-C, and dyslipidemia, and the OR ranged from 1.28 to 2.25. However, the statistically significant associations were observed only for high TG, low HDL-C and dyslipidemia among females, and the OR is lower than these for males in all types of dyslipidemia. An interactive effect of hypertension and general or central obesity on low HDL-C, high TG and dyslipidemia was observed, but not in high TC and LDL-C (*p* for interaction > 0.05). Among participants without hypertension, the stronger associations were found between general obesity and low HDL-C, high TG, and dyslipidemia, with OR being 2.21, 2.16 and 2.10, respectively. Similar associations were also observed between central obesity and high TG, low HDL-C, and dyslipidemia. With subgroup analysis by diabetes, no effect modification was observed on the associations between obesity and high TG, high TC, and high LDL-C. Ann interaction of diabetes and general obesity on low HDL-C only was shown (*p* for interaction = 0.007), while the interaction of diabetes and central obesity on high TG, dyslipidemia was found (*p* for interaction < 0.05). 

## 4. Discussion

This study aimed to comprehensively examine the positive associations of obesity and TC, TG and LDL-C levels, as well as the negative associations of obesity with HDL-C levels in a suburban area experiencing rapid urbanization of China. Though previous studies have been conducted on obesity or serum lipid, few studies have explored the association between obesity and various types of dyslipidemia concurrently in an area that has rapidly urbanized in China. Our results indicated that the prevalence of general obesity and central obesity were 13.5% and 28.9%, which was in line with previous large-scale surveys among Chinese [25,26]. Dyslipidemia was prevalent in most obese subjects and revealed nearly half of general obesity participants suffered dyslipidemia and higher values of LDL-C, TG, TC, and lower HDL-C than in normal-weight individuals [27]. Several previous regional epidemiological studies reported the prevalent dyslipidemia in obesity subjects differently [28,29]. The possible reasons for this discrepancy might be the socio-demographic characteristics of the subjects and the diagnostic criteria used [30]. Moreover, the differences in dietary pattern may also play a role in regional differences in the prevalence of dyslipidemia [31]. 

A recent study found that the incidence of deaths and disability-adjusted life years (DALYs) attributable to obesity has increased significantly [32]. Several studies have demonstrated that overweight and obesity are cardiometabolic risk factors [33,34,35]. Data from 97 prospective cohorts with 1.8 million participants indicate that obesity is associated with 31% coronary heart disease risk and 8% stroke mortality risk, due to elevated blood pressure and cholesterol together [36]. Therefore, effective control of lipid level can be expected to attenuate death from metabolic diseases. This study found that general or central obesity were associated with higher prevalence of dyslipidemia compared with non-obesity. The major types of dyslipidemia among obesity subjects are low HDL-C and high TG, which is consistent with other research and probably due to the elevated TG and Apo lipoprotein B from excess visceral fat in the abdomen and a low HDL-C production from inhibition of the liver [13,37]. A strong association has been identified between central obesity and metabolic risk factors, cardiovascular events and dyslipidemia [38]. As we know, higher levels of BMI and WC correlate with increased prevalence of abnormal lipids, depending on gender and age. It is clear that a higher BMI and WC contribute to the development of these metabolic diseases.

Our study suggested that subjects with obesity either by BMI or WC had higher risk of dyslipidemia than those without obesity, which were comparable to those of previous studies [39,40]. The adjusted multivariate logistic regression shows that the effect is the highest between general or central obesity and low HDL-C; in addition, the ORs between the WC and dyslipidemia are higher than that of BMI and dyslipidemia, indicating that WC has a greater influence on lipid level than BMI. Central obesity characterized by the accumulation of visceral fat in the abdomen is more closely associated with a global metabolic effect of insulin resistance than general obesity [41,42]. Insulin resistance might change the amount and composition of lipoprotein to cause abnormal blood lipid levels, which act on the metabolism of LDL, chylomicron, HDL, and very-low-density lipoprotein (VLDL) [43]. 

The modifying effects of gender, age, hypertension, and diabetes on the association between obesity and dyslipidemia were further observed. Compared with females, significantly higher risk of different forms of dyslipidemia was observed in obese males. A possible reason for this is the intensity of work pressure, unhealthy lifestyle and diets for males, which account for excessive fat accumulation [44]. The proportion of smoking and drinking among males is higher than that of females, which are risk factors of dyslipidemia [45]. Obese subjects younger than 60 years were at greater risk of different forms of dyslipidemia. Therefore, more targeted prevention should be formulated for residents, adapting to different genders and age groups. Hypertension exerted an effect modification on the association of obesity with low HDL-C, high TG and dyslipidemia. Non-hypertensive obese individuals may be more prone to low HDL-C, high TG and dyslipidemia. Similarly, diabetes exerted an effect modification on the association of general obesity with low HDL-C and the association of central obesity with high TG, dyslipidemia. However, the causality among obesity, hypertension, diabetes, and dyslipidemia remains association these may exert a comprehensive effect on each other. A previous study found that obesity can be caused by dyslipidemia and subjects with dyslipidemia are more likely to experience hypertension [46]. Other studies showed that weight gain, elevated blood pressure and blood glucose may be essential in incident dyslipidemia [47,48]. In short, we propose that effectively management of BMI or WC would be helpful in preventing and controlling different forms of dyslipidemia.

In this study, the main strengths included a large sample size and stratified analysis on the association between obesity and different types of dyslipidemia. We additionally considered the possible confounding effect of dietary quality on the associations and adjusted the CHDI in the final model. However, a few limitations should be taken into consideration. Firstly, the study is a cross-sectional study in Shanghai, which cannot provide causal relationships and may not be generalizable to different geographical regions. Thus, further prospective studies are necessary to verify the relationship of different indices of adiposity with dyslipidemia. Second, potential residual confounding factors (such as stress, sleep pattern, genetic factors) associated with dyslipidemia could not be considered, which seem to bias the results. In addition, the possible confounding factors of the lean and fat mass percentages or ratios that are directly connected to the LDL and TG levels were not considered because of limitations in budget and equipment. Nevertheless, BMI and WC are the most readily available indicators to estimate obesity. We did not account for the impact of these indicators (blood glucose, insulin levels, adiponectin, leptins) on dyslipidemia and may underestimate the prevalence of dyslipidemia. However, diabetes diagnosed based on FPG was adjusted in the models. Finally, health-related behaviors were reported by the participants themselves and we could not rule out subjects’ error, which may lead to a reporting bias.

## 5. Conclusions

In conclusion, our study found that general or central obesity have a higher prevalence of different forms of dyslipidemia, and the major types were low HDL-C and high TG among adults in Shanghai. Central obesity may have a greater effect on lipid level than general obesity. Factors including age, gender, hypertension, and diabetes might be potential modifiers of the association. A significantly higher OR of various types of dyslipidemia was observed in obese younger than 60 years, males, without hypertension and diabetes. Effective management of obesity should be implemented to prevent and control the occurrence of dyslipidemia.

## Figures and Tables

**Table 1:** Basic characteristics of the participants based on general and central obesity.

Characteristics		BMI		*p*	WC		*p*
	All Subjects (*n* = 40,406)	Non-General Obesity (*n* = 35,207)	General Obesity ^a^ (*n* = 5199)		Non-Central Obesity (n = 28,761)	Central Obesity ^b^ (n = 11,645)	
Age (years)	56 ± 11	56 ± 11	57 ± 10	<0.001	55 ± 11	59 ± 10	<0.001
Gender				<0.001			<0.001
Male	16,793 (41.6)	14,471 (41.1)	2322 (44.7)		11,789 (41.0)	5004 (43.0)	
Female	23,613 (58.4)	20,736 (58.9)	2877 (55.3)		16,972 (59.0)	6641 (57.0)	
Education level				<0.001			<0.001
0–6 years	16,557 (41.0)	14,072 (40.0)	2485 (47.8)		10,823 (37.6)	5734 (49.2)	
7–12 years	21,017 (52.0)	18,561 (52.7)	2456 (47.2)		15,561 (54.1)	5456 (46.9)	
>12 years	2832 (7.0)	2574 (7.3)	258 (5.0)		2377 (8.3)	455 (3.9)	
Marriage				0.73			0.61
Married	37,584 (93.0)	32,754 (93.0)	4830 (92.9)		26,764 (93.1)	10,820 (92.9)	
Other	2822 (7.0)	2453 (7.0)	369 (7.1)		1997 (6.9)	825 (7.1)	
Physical activity				0.38			<0.001
Low	13,237 (32.8)	11,574 (32.9)	1663 (32.0)		9587 (33.3)	3650 (31.3)	
Moderate	13,771 (34.1)	11,994 (34.1)	1777 (34.2)		9840 (34.2)	3931 (33.8)	
High	13,398 (33.2)	11,639 (33.1)	1759 (33.8)		9334 (32.5)	4064 (34.9)	
Smoking				0.14			0.003
Ever	10,082 (25.0)	8742 (24.8)	1340 (25.8)		7059 (24.5)	3023 (26)	
Never	30,324 (75.1)	26,465 (75.2)	3859 (74.2)		21,702 (75.5)	8622 (74)	
Alcohol drinking				0.002			<0.001
Ever	5563 (13.8)	4774 (13.6)	789 (15.2)		3758 (13.1)	1805 (15.5)	
Never	34,843 (86.2)	30,433 (86.4)	4410 (84.8)		25,003 (86.9)	9840 (84.5)	
Diabetes				<0.001			<0.001
Yes	5738 (14.2)	4420 (12.6)	1318 (25.4)		3154 (11.0)	2584 (22.2)	
No	34,668 (85.8)	30,787 (87.5)	3881 (74.7)		25,607 (89)	9061 (77.8)	
Hypertension				<0.001			<0.001
Yes	19,696 (48.8)	15,948 (45.3)	3748 (72.1)		11,227 (45.0)	7102 (70.2)	
No	20,710 (51.3)	19,259 (54.7)	1451 (27.9)		16,769 (58.3)	3941 (33.8)	
CHDI	70.05 ± 9.29	70.2 ± 9.31	69.04 ± 9.13	<0.001	70.51 ± 9.28	68.91 ± 9.22	<0.001
HBA1c (%)	4.9 (4.4, 5.5)	4.8 (4.4, 5.4)	5.1 (4.5, 5.9)	<0.001	4.8 (4.4, 5.4)	5.0 (4.4, 5.8)	<0.001
FPG (mmol/L)	5.81 ± 0.85	5.77 ± 0.81	6.09 ± 1.00	<0.001	5.72 ± 0.78	6.03 ± 0.97	<0.001
TC (mmol/L)	4.91 ± 0.94	4.90 ± 0.93	4.99 ± 0.99	<0.001	4.88 ± 0.92	4.99 ± 0.97	<0.001
TG (mmol/L)	1.70 ± 1.26	1.64 ± 1.2	2.11 ± 1.55	<0.001	1.56 ± 1.12	2.03 ± 1.49	<0.001
HDL-C(mmol/L)	1.4 ± 0.35	1.42 ± 0.35	1.26 ± 0.31	<0.001	1.44 ± 0.35	1.29 ± 0.32	<0.001
LDL-C (mmol/L)	2.74 ± 0.83	2.74 ± 0.83	2.78 ± 0.88	0.004	2.73 ± 0.82	2.78 ± 0.87	<0.001

^a^ General obesity defined as BMI ≥ 28.0 kg/m^2^, ^b^ central obesity defined as WC ≥ 90 cm in males and ≥85 cm in females.

**Table 2:** Stratified analysis of general obesity with dyslipidemia.

Variables	High TC	High LDL-C	Low HDL-C	High TG	Dyslipidemia
Age (years)					
<60	1.26 (1.09, 1.45)	1.28 (1.07, 1.52)	1.87 (1.68, 2.07)	1.81 (1.65, 1.99)	1.88 (1.72, 2.05)
≥60	0.97 (0.84, 1.13)	0.89 (0.74, 1.07)	1.66 (1.49, 1.84)	1.65 (1.49, 1.82)	1.60 (1.47, 1.75)
*p* for Interaction	<0.001	<0.001	0.14	0.001	<0.001
Gender					
Male	1.53 (1.29, 1.81)	1.41 (1.14, 1.74)	1.94 (1.76, 2.14)	2.05 (1.85, 2.26)	2.24 (2.04, 2.46)
Female	0.90 (0.79, 1.02)	0.92 (0.78, 1.08)	1.48 (1.31, 1.67)	1.45 (1.31, 1.59)	1.40 (1.29, 1.53)
*p* for Interaction	<0.001	<0.001	0.01	<0.001	<0.001
Hypertension					
Yes	1.05 (0.94, 1.19)	1.05 (0.90, 1.21)	1.63 (1.49, 1.78)	1.63 (1.50, 1.76)	1.65 (1.53, 1.77)
No	1.25 (1.02, 1.53)	1.19 (0.93, 1.53)	2.21 (1.93, 2.54)	2.16 (1.90, 2.46)	2.10 (1.88, 2.34)
*p* for Interaction	0.15	0.40	<0.001	<0.001	0.001
Diabetes					
Yes	1.03 (0.85, 1.26)	1.07 (0.83, 1.37)	1.50 (1.30, 1.74)	1.62 (1.42, 1.85)	1.63 (1.43, 1.86)
No	1.13 (1.01, 1.28)	1.10 (0.94, 1.27)	1.89 (1.74, 2.06)	1.83 (1.69, 1.98)	1.83 (1.71, 1.96)
*p* for Interaction	0.46	0.84	0.007	0.06	0.17

Adjusted for gender, age, education level, marriage, physical activity, smoking, alcohol drinking, CHDI, diabetes, and hypertension, except for a stratifying variable.

**Table 3:** Stratified analysis of central obesity with dyslipidemia.

Variables	High TC	High LDL-C	Low HDL-C	High TG	Dyslipidemia
Age (years)					
<60	1.23 (1.10, 1.38)	1.37 (1.19, 1.58)	2.04 (1.87, 2.21)	2.13 (1.98, 2.30)	2.10 (1.96, 2.24)
≥60	1.02 (0.91, 1.13)	0.97 (0.85, 1.11)	1.68 (1.55, 1.83)	1.70 (1.57, 1.85)	1.64 (1.53, 1.75)
*p* for Interaction	<0.001	<0.001	0.008	<0.001	<0.001
Gender					
Male	1.31 (1.14, 1.51)	1.28 (1.08, 1.52)	2.03 (1.88, 2.19)	2.22 (2.05, 2.40)	2.25 (2.10, 2.42)
Female	0.99 (0.90, 1.09)	1.04 (0.93, 1.17)	1.60 (1.45, 1.76)	1.64 (1.51, 1.76)	1.52 (1.43, 1.62)
*p* for Interaction	0.003	0.09	0.15	0.04	<0.001
Hypertension					
Yes	1.11 (1.01, 1.22)	1.10 (0.98, 1.25)	1.75 (1.63, 1.89)	1.79 (1.67, 1.92)	1.75 (1.65, 1.85)
No	1.17 (1.02, 1.33)	1.24(1.06, 1.46)	2.20 (2.00, 2.42)	2.41 (2.20, 2.63)	2.18 (2.02, 2.35)
*p* for Interaction	0.11	0.09	<0.001	<0.001	<0.001
Diabetes					
Yes	1.08 (0.91, 1.28)	1.09 (0.88, 1.36)	1.76 (1.54, 2.00)	1.73 (1.54, 1.95)	1.74 (1.56, 1.94)
No	1.13 (1.04, 1.24)	1.16 (1.04, 1.30)	1.96 (1.83, 2.09)	2.07 (1.94, 2.20)	1.94 (1.85, 2.04)
*p* for Interaction	0.34	0.47	0.12	0.001	0.048

Adjusted for gender, age, education level, marriage, physical activity, smoking, alcohol drinking, CHDI, diabetes, and hypertension, except for a stratifying variable.

## Data Availability

The dataset used and analyzed during the current study is available from the corresponding author upon reasonable request.

## Abbreviations

SSACB	The Shanghai Suburban Adult Cohort and Biobank study
OR	Odds ratios
CI	Confidence interval
TC	Total cholesterol
TG	Triglycerides
LDL-C	Low-density lipoprotein cholesterol
HDL-C	High-density lipoprotein cholesterol
WC	Waist circumference
FPG	Fasting plasma glucose
HbA1c	Glycated hemoglobin
BMI	Body mass index
SBP	Systolic blood pressure
DBP	Diastolic blood pressure
ADA	American Diabetes Association
CHDI	China Healthy Diet Index
MET	Metabolic equivalent of task
SD	Standard deviation
IQR	Interquartile range
DALYs	Disability-adjusted life years
